# Contralateral Suppression of DPOAEs in Mice after Ouabain Treatment

**DOI:** 10.1155/2018/6890613

**Published:** 2018-04-10

**Authors:** Jieying Li, Yan Chen, Shan Zeng, Chuijin Lai, Yanping Zhang, Liting Zhang, Yuxuan Shi, Tianyu Zhang, Huawei Li, Peidong Dai

**Affiliations:** ^1^ENT Institute and Otorhinolaryngology Department of Affiliated Eye and ENT Hospital, State Key Laboratory of Medical Neurobiology, Fudan University, Shanghai 200031, China; ^2^Key Laboratory of Hearing Medicine of NHFPC, Shanghai 200031, China; ^3^Department of Cardiology, Shandong Provincial Hospital Affiliated to Shandong University, 324 Jing Wu Road, Shandong, China; ^4^Institutes of Biomedical Sciences, The Institutes of Brain Science and the Collaborative Innovation Center for Brain Science, Fudan University, Shanghai 200032, China; ^5^Shanghai Engineering Research Centre of Cochlear Implant, Shanghai 200031, China

## Abstract

Medial olivocochlear (MOC) efferent feedback is suggested to protect the ear from acoustic injury and to increase its ability to discriminate sounds against a noisy background. We investigated whether type II spiral ganglion neurons participate in the contralateral suppression of the MOC reflex. The application of ouabain to the round window of the mouse cochlea selectively induced the apoptosis of the type I spiral ganglion neurons, left the peripherin-immunopositive type II spiral ganglion neurons intact, and did not affect outer hairs, as evidenced by the maintenance of the distorted product otoacoustic emissions (DPOAEs). With the ouabain treatment, the threshold of the auditory brainstem response increased significantly and the amplitude of wave I decreased significantly in the ouabain-treated ears, consistent with the loss of type I neurons. Contralateral suppression was measured as reduction in the amplitude of the 2f_1_−f_2_ DPOAEs when noise was presented to the opposite ear. Despite the loss of all the type I spiral ganglion neurons, virtually, the amplitude of the contralateral suppression was not significantly different from the control when the suppressor noise was delivered to the treated cochlea. These results are consistent with the type II spiral ganglion neurons providing the sensory input driving contralateral suppression of the MOC reflex.

## 1. Introduction

In the cochlea, the neurons of the spiral ganglion emit peripheral processes that extend to the organ of Corti and have central processes that project to the cochlear nuclei via the auditory nerve. There are two types of spiral ganglion neurons (SGNs): (1) myelinated type I spiral ganglion cells, which innervate the inner hair cells and represent 90%–95% of the population; (2) unmyelinated type II ganglion cells, which innervate the outer hair cells (OHCs) and represent 5%–10% of the population. The scarcity and small caliber axons of the type II SGNs make them difficult to study. In consequence, there has been little experimental research into the physiology of the type II spiral SGNs [1].

To magnify low-intensity sounds and compress the dynamic intensity range, cochlear outer hair cells amplify the basilar membrane vibrations in a nonlinear, intensity-dependent pattern. Instantaneous waveform distortion is produced in this process, generating new sound frequencies that are absent from the original stimulation. These distortion products can be detected in the ear canal as otoacoustic emissions. The “cochlear amplifier” can be evaluated by measuring the distortion product otoacoustic emissions (DPOAEs) [2].

Medial olivocochlear (MOC) fibers projecting to the cochlea originate on both sides of the medial portion of the superior olivary complex, where they form synapses with the outer hair cells (OHCs). By hyperpolarizing the OHCs, the MOC efferents inhibit the electromotility of the OHCs, thereby reducing the gain of the cochlear amplifier, which manifests as a reduction in the DPOAEs. The MOC reflex includes both contralateral suppression and ipsilateral suppression. The contralateral suppression is commonly detected with a suppressor sound contralateral to the DPOAE test ear [3]. With an intact olivocochlear bundle, the amplitude of DPOAEs decrease quickly after the contralateral noise is turned on. After this “rapid onset adaptation,” the response returns to a value similar to that before the contralateral noise [3]. MOC efferent feedback is suggested to protect the ear from acoustic injury and to increase its ability to discriminate sounds against a noisy background [3].

However, the sensory input that drives the MOC efferent reflex has not been identified. A recent study [4], based on a peripherin (*Prph*) knockout mouse model, proposed that the type II SGNs drive the MOC reflex. Peripherin is strongly expressed in type II (but not type I) SGNs [5]. In the transgenic mouse model, the outer spiral bundle of type II SGNs is largely absent in *Prph* ^−/−^ cochleae [4]. The study found that both contralateral suppression and ipsilateral suppression were almost totally lost in the *Prph* ^−/−^ ears compared with *Prph* ^+/+^ ears. On the contrary, another report suggested that type II SGNs are not the sensory limb of the cochlear efferent reflex [6]. That study showed that peripherin is also expressed in MOC fibers and that the inactivation of the MOC reflex observed in the peripherin knockout mice could be interpreted as the loss of the MOC function [6]. Therefore, in this study, an ouabain-treated mouse model was used, in which the apoptosis of the type I SGNs was induced, while the type II SGNs remained intact [7–9], to explore whether the type II SGNs play a role in the MOC reflex.

## 2. Materials and Methods

### 2.1. Animal Groups and Surgery

The cold method was used to prepare poloxamer 407 gels [10]. Poloxamer 407 was slowly added to cold distilled water to prepare a 20% (weight/weight) stock solution. The ouabain solution was prepared by dissolving ouabain powder in distilled water to produce a 10 mM stock solution. The stock solution was diluted with cold distilled water to produce a solution containing 18% (weight/weight) poloxamer 407 and 2 mM ouabain. This formulation is in a fluid state at room temperature and in the gel state at the body temperature of mice. The gel promotes the prolonged release of ouabain.

The experiments were performed on eight 4-week-old male C57BL/6 mice. The animals were anesthetized with pentobarbital sodium (50 mg/kg, intraperitoneally (i.p.)). One-third of the initial dose of anesthetic was given when needed. A posteroinferior skin incision was made in the retroauricular area of the right ear. To expose the bulla, the nearby muscles and facial nerve were separated. A small opening was made in the bulla to expose the round window. A 2.5 *μ*l Hamilton syringe was used to apply the ouabain gel described above (1.6 *μ*l) to the round window membrane. Sham operations were conducted in another group of 4-week-old C57BL/6 mice as controls (*n* = 8). The bulla was covered with bone wax, and a nonabsorbable suture was used to close the incision. The animal was placed on a homeothermic blanket for recovery. Electrophysiological tests of cochlear function were conducted 2 weeks after the surgeries. All the procedures were approved by the Institutional Animal Care and Use Committee of the Eye and ENT Hospital of Fudan University, China.

### 2.2. Cochlear Function Tests

An auditory-evoked potential and DPOAE workstation (TDT system 3 with RX6 and RX6-2 signal processors; Tucker Davis Technologies, Fort Lauderdale, FL, USA) was used to conduct the cochlear function tests, with the BioSig32 software. The auditory brainstem responses (ABRs) and DPOAEs were recorded as per previous study [4]. The amplitude of the 2f_1_−f_2_ (cubic) distortion products and the surrounding noise floor were recorded. The mice were anesthetized with ketamine (100 mg/kg, i.p.) and xylazine (10 mg/kg, i.p.). The threshold for ABR was determined as the lowest intensity at which a repeatable ABR waveform could be identified. The wave I component was identified and the peak-to-peak amplitude computed by off-line analysis of stored waveforms. In the DPOAE recordings, the frequency ratio f_2_/f_1_ was 1.2 and the intensity of f_1_ and f_2_ was the same, increasing together in 5 dB steps (from 20 dB SPL to 80 dB SPL). The amplitude of the 2f_1_−f_2_ distortion products and the surrounding noise floor were recorded. The DPOAE thresholds were determined based on when the cubic (2f_1_–f_2_) distortion product reached 5 dB above the noise floor, as the tone intensity increased, as previously described [4].

The contralateral suppression was measured after DPOAE test without dismounting the DPOAE-measuring probe from the untreated ear. The parameter setting for contralateral suppression was optimized based on prior studies [11, 12]. The right ears (after surgeries) of ouabain-treated mice were exposed to 76 dB SPL, 13–20 kHz broadband suppressor noise (continuous for 15 s, closed field), whereas the DPOAEs were elicited in the left ears (without surgeries) with 60 dB SPL, 16 kHz primary tones. Sham-operated mice without ouabain treatment served as controls. Three measurements were averaged (4/s) for each recording. The DPOAEs were monitored before and after the suppression noise to obtain the baseline DPOAE measurement. The amplitude of the 2f_1_−f_2_distortion products relative to the noise floor was recorded before, during, and after the noise stimulation. The broadband noise was generated with the Cool Edit Pro software (Adobe Systems, San Jose, CA, USA). The stimulus was delivered as closed field using MF1 Multi-Field Magnetic Speakers (Tucker-Davis Technologies), with a customized coupler. To address the effect of the cross talk to contralateral suppression, we measured contra-noise effects before and after mechanical destruction of the contralateral cochlea (right ears) by opening the cochlear basal turn (*n* = 6). As shown in the Supplemental Figure 1, the destruction completely eliminated the contralateral suppression.

### 2.3. Immunofluorescence Staining

Immediately after the dissection of the cochlea, 4% paraformaldehyde in phosphate-buffered saline (PBS) was perfused through the round window and oval window. The cochlea was immersed in the same solution for 2 h at 4°C. For frozen sectioning, the cochlea was decalcified (0.1 M ethylenediaminetetraacetic acid (EDTA)) and cryoprotected in 30% sucrose at 4°C overnight prior to embedding in optimal cutting temperature compound (OCT). Then, the sections were made and mounted on slides for immunofluorescence staining. Immunostaining began with blocking buffer (10% donkey serum in PBS) for 2 h at room temperature. The sections were then incubated at 4°C overnight in a combination of the following primary antibodies: (1) mouse anti-tubulin *β*3 (Tuj1, BioLegend), diluted 1 : 300; (2) rabbit antiperipherin (Prph, Abcam), diluted 1 : 500. The samples were then incubated at 4°C overnight with a species-appropriate secondary antibody (Alexa Fluor 488-labeled anti-mouse IgG antibody, Alexa Fluor 594-labeled anti-rabbit IgG antibody). The images were captured using fluorescence microscope (NIKON ECLIPSE Ni U, Nikon Instruments Inc., Japan), and Photoshop CS5 software (Adobe Systems, San Jose, CA, USA) was used to adjust contrast and brightness of images.

### 2.4. SGN Counting

Tubulin *β*3- (Tuj1-) positive and peripherin- (Prph-) positive neurons in 12 serial midmodiolar sections of the cochlear (120 *μ*m thickness in total) were counted [13]. The sectional area of Rosenthal's canal was calculated from the images with the Photoshop CS5 software (Adobe Systems, San Jose, CA, USA) as the literature [13] described; an image of a standard slide was used to calibrate the scale, which was converted from pixels to micrometers; the outline of Rosenthal's canal was circumscribed in every section; the total number of pixels for Rosenthal's canal was calculated and converted into square micrometers. In Rosenthal's canal, Tuj1-positive and Prph-negative cells were defined as type I SGNs; Tuj1-positive and Prph-positive cells were defined as type II SGNs. The total number of each type of SGN was then divided by the two-dimensional area to obtain the density of SGNs per square millimeter [14].

### 2.5. Statistical Analysis

**Figure 1:**
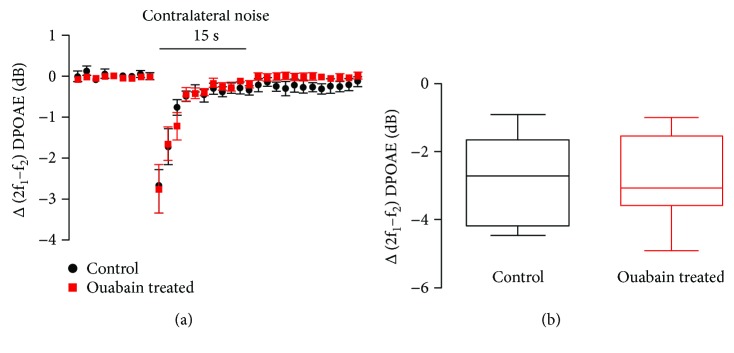
Ouabain treatment did not significantly alter contralateral suppression. (a) The time course of contralateral suppression showed no significant difference between the control and ouabain-treated groups. *N* = 8, data were shown as the mean ± SEM. (b) The amplitude of the peak suppression of the control and ouabain-treated groups. Fifteen seconds of 76 dB SPL. 13–20 kHz noise produced a similar reduction in the amplitude of the 2f_1_−f_2_ DPOAE (60 dB SPL 16 kHz) in the control and ouabain-treated ears. Data in (b) showed the amplitude of the peak suppression which is from the first measurement after noise onset relative to the average of the prenoise baseline. *N* = 8, the boundaries indicated 25th and 75th percentile; solid line indicated median; error bars indicated the maximum and minimum.

Data are expressed as the population mean ± standard errors of the means (SEM) except for the data in Figure 1(b). The statistical analysis was performed with SPSS 11.5 (SPAA Inc., Chicago, IL, USA). Two-way ANOVA was used to compare the thresholds of ABR and DPOAE and the amplitudes of the 2f_1_−f_2_ distortion products at 16 kHz and the amplitude of wave I between the ouabain-treated and control groups [4]. Two-way ANOVA was also used to compare contralateral suppression between the ouabain-treated and control groups [4]. One-way ANOVA was used to compare the peak amplitudes of the rapid contralateral suppression between the ouabain-treated and control groups and to compare the numbers of type I and type II SGNs between the ouabain-treated and control groups.

## 3. Results

### 3.1. Cochlear Function Tests

**Figure 2:**
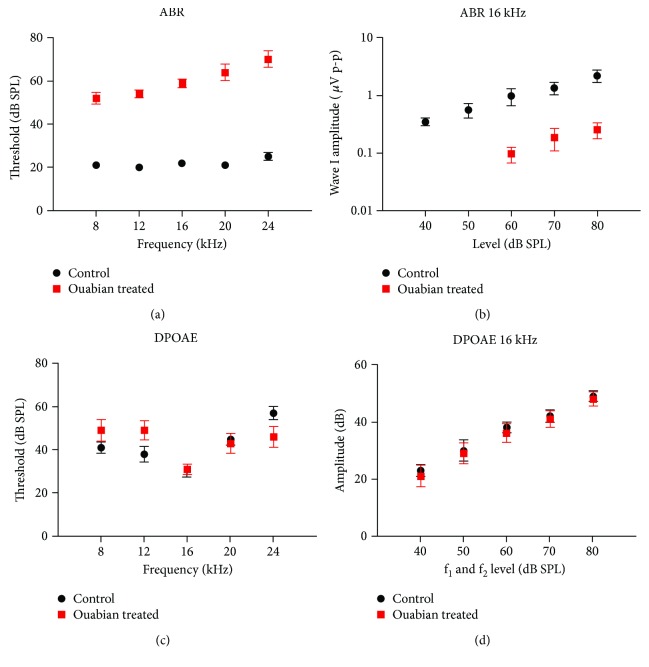
ABR and DPOAE results of the control and ouabain-treated ears. (a) ABR thresholds for the control ears (*n* = 8) versus ouabain-treated ears (*n* = 8) at 2 weeks after the application of ouabain. (b) Mean amplitudes of ABR wave I at 16 kHz for the animals shown in (a). (c) DPOAE thresholds for the animals shown in (a). (d) Mean amplitudes versus level functions at f_2_ = 16 kHz for the animals shown in (a). *N* = 8, data were shown as the mean ± SEM.

To explore whether type II spiral ganglion neurons participate in the contralateral suppression of the MOC reflex, ouabain was applied to the right cochlea at the round window to eliminate the type I spiral ganglion neurons. This application of ouabain selectively induced the apoptosis of the type I spiral ganglion neurons, but left the type II spiral ganglion neurons intact as reported previously [8]. The sham-operated mice without ouabain treatment were used as controls. Cochlear function was tested after the ouabain treatment. Two weeks after the application of ouabain, the ABR threshold increased significantly compared with that in the control ear (*P* < 0.01 at 8, 12, 16, 20, and 24 kHz; Figure 2(a)). The ABR thresholds increased by 30–45 dB SPL at all frequencies after ouabain treatment. Besides, the amplitude of wave I at 16 kHz decreased significantly compared with the control group (*P* < 0.01 at 60, 70, and 80 dB SPL, Figure 2(b)). However, ouabain treatment had no significant effect on the threshold and amplitude of DPOAEs at 16 kHz compared with the control ears (Figures 2(c) and 2(d)), indicating that the cochlear outer hair cells remained intact 2 weeks after the application of ouabain.

### 3.2. Cochlear Histopathology

**Figure 3:**
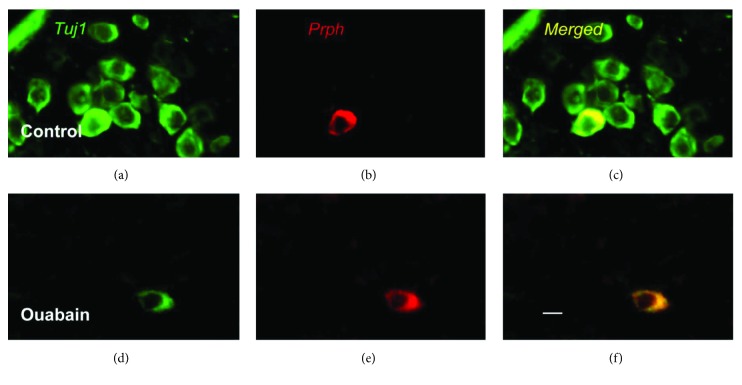
Images of spiral ganglion neurons in control and ouabain-treated ears. (a–c) Representative image of Tuj1 and Prph immunostaining in spiral ganglion cells of the control group. Prph, a marker of type II spiral ganglion cells, was detected with Alexa Fluor 594 (red). Tuj1, a marker of type I and type II neurons, was detected with Alexa Fluor 488 (green). (d–f) Representative image of Tuj1 and Prph immunostaining in spiral ganglion cells of the ouabain-treated group. Nearly, all type I spiral ganglion cells were lost, whereas Prph-positive type II neurons survived after ouabain exposure. Scale bar = 20 *μ*m.

We immunostained frozen sections of the whole cochleae for two marker proteins: (1) tubulin *β*3 (Tuj1), which is expressed in both type I and type II SGNs, and (2) peripherin (Prph), a type III intermediate filament, whose immunoreactivity is restricted to the soma and processes of type II SGNs in the mature cochlea. Figures 3(a) and 3(d) showed that after ouabain treatment, there was a nearly complete elimination of type I SGCs. Figures 3(b) and 3(e) show that nearly all of peripherin-positive type II SGCs remained, which were detected in both the control cochleae and the experimental cochleae 2 weeks after treatment with ouabain.

### 3.3. SGN Counting

The type I SGNs (Tuj1-positive and Prph-negative) and type II SGNs (Tuj1-positive and Prph-positive) were counted in 12 midmodiolar sections of the ouabain-treated and control cochleae. In the control group, the average densities of type I and type II SGNs were 1898 ± 103/mm^2^ and 202 ± 58/mm^2^, respectively. After ouabain treatment, about 99% of the type I SGNs were eliminated and the density of the remaining type I neurons was 10 ± 6/mm^2^ (*P* < 0.01). Meanwhile, the average density of surviving type II SGNs was 190 ± 62/mm^2^. The density of type II SGNs has no significant difference between the control and ouabain treatment groups (*P* = 0.97). These data indicate that there was no loss of type II neurons and almost total loss of type I neurons after the application of ouabain. These results also suggest that about 9.59% ± 0.39% of the SGNs in the normal mouse ear were type II.

### 3.4. Contralateral Suppression in Mice of Control and Ouabain-Treated Groups

The functional effect of the MOC reflex, evident as contralateral suppression, was evaluated as a reduction in 2f_1_−f_2_ DPOAEs induced by suppressor noise in the ear opposite to that which the DPOAE was measured in. With an intact olivocochlear bundle, the amplitude of distortion products decreased quickly after the contralateral noise was turned on. Thus, wideband noise was delivered to the ouabain-treated ears (right ears) and the DPOAEs were recorded in the left ears as the previous study reported [15]. Sham-operated mice without ouabain treatment served as controls. Both in the control and ouabain-treated groups, suppression was obvious from the onset of the contralateral noise, and the maximal reduction in the DPOAEs occurred within 1 s (Figure 1(a)). The suppression displayed near-complete adaptation after the noise had been presented for 10 s. The ouabain-treated ears showed similar contralateral suppression time course to that in the control group (Figure 1(a), *P* = 0.35). The amplitude of the peak suppression in the ouabain-treated ears (2.88 ± 0.46 dB, *n* = 8) did not differ significantly from that in the control group (2.86 ± 0.46 dB, *n* = 8, Figure 1(b), *P* = 0.77). The peak suppression in both the control and ouabain-treated ears occurred within 1 s after the onset of the contralateral noise. The control and ouabain-treated ears showed similar rates of shift in their DPOAEs.

## 4. Discussion

The afferents of the MOC reflex must originate from either the type I SGNs and/or the type II SGNs. A recent report [4] proposed that the type II neurons drive the MOC reflex. A peripherin knockout mouse model lacking type II innervation of the cochlea was used in that study. Peripherin is strongly expressed in type II but not type I SGNs [5]. The contralateral and ipsilateral suppression of the MOC reflex was nearly eliminated in the *Prph* ^−/−^ ears compared with that in the *Prph* ^+/+^ ears. On the contrary, another report suggested that peripherin is also expressed in some MOC fibers. The loss of MOC function in the peripherin knockout mice can be interpreted as the inactivation of contralateral suppression and ipsilateral suppression [6]. Besides the type II neurons, the function of other peripheral neurons is probably affected in *Prph* ^−/−^ mice because peripherin is widely expressed in the peripheral nervous system. To study the function of type II neurons, an animal model that separates type I and type II neurons without affecting other neurons is required. Therefore, in this study, we used a mouse model in which ouabain was applied unilaterally to the cochlear round window, which induced the apoptosis of type I SGNs but left the type II SGNs intact in the ouabain-treated ear. The MOC fibers in the other ear (in which DPOAE were elicited) were not affected by ouabain. Contralateral suppression was used to evaluate the inhibition of cochlear amplification by the MOC reflex.

Several previous studies [7, 8, 16, 17] have shown that the application of ouabain to the round window causes the nearly complete elimination of the type I SGNs but leaves the type II SGNs intact, with no obvious loss of or damage to the hair cells. The results of our electrophysiological tests of cochlear function and cochlear histopathology in the ouabain-treated ears are consistent with those of the previous studies. Ouabain treatment significantly elevated the ABR threshold and decreased the amplitude of wave I, with no significant shift in the DPOAE threshold or amplitude (Figure 2). Moreover, the immunofluorescence staining further confirmed that the application of ouabain selectively causes the death of type I neurons, without damaging type II neurons (Figure 3).

After ouabain exposure, the amplitude of contralateral suppression was not significantly different from that in the control group. Because the application of ouabain to the round window of the cochlea induces the apoptosis of type I SGNs but leaves the type II SGNs intact [7–9, 16], the pathway from the inner hair cells and their type I SGNs to the brain is almost silenced in the ouabain-treated ears. Therefore, the responses of these ears to sound are attributed to an alternative mechanism of auditory sensing in which the outer hair cells and type II SGNs probably participate. The afferents of the MOC suppression reflex must either be type I SGNs and/or type II SGNs. Our results support the hypothesis that type II SGNs exclusively drive the MOC contralateral suppression reflex.

Recent studies suggest that type II SGNs act as cochlear nociceptors, functioning only when the OHCs are damaged [18, 19]. Cochlear type II afferents and somatosensory C-fiber nociceptors share anatomical features, physiological properties, and protein expression [19]. Maison et al. [6] proposes that this finding contradicts the hypothesis that the type II SGNs drive the MOC contralateral suppression reflex, because nociceptors respond to traumatically high sound levels, whereas cochlear efferents respond near the hearing threshold. However, Flores et al. speculates that instead of pain, auditory nociception might elicit an axon reflex, an autonomic reaction, or an efferent response, like MOC reflex, to protect the inner ear from further damage [19].

There is only a 10–20 dB difference between the thresholds of MOC neurons and type I neurons [20], and type II neurons must be similarly sensitive to sound if they act as the afferents of this reflex. Recent electrophysiological experiments in isolated rat organs of Corti support this hypothesis. The integration of synaptic input from multiple OHCs by type II SGNs was identified [21]. The length constants of type II SGNs imply that synaptic inputs can sum effectively through the processes of the type II neurons [21]. Experimental records and the computational model both imply that the extended dendrites of the type II neurons can integrate these inputs. It is speculated that the small synaptic inputs elicited by the neurotransmitters released by individual OHCs will sum approximately linearly across the many tens of OHCs innervated by each of the type II SGN neurites. In the computation model, six synchronous synaptic inputs are required to generate a spike. Because the probability of transmitter release by OHCs is low, the simultaneous stimulation of 24 OHCs is required for action potential in the model of type II SGNs. This number is in the range of OHCs connected to type II neurons, estimated in the previous studies [22].

Some studies have reported that type II SGNs are insensitive to sound [23–25]. However, the antidromic stimulation used in those studies may not reflect the physical electrical features of type II SGNs. Moreover, the sample sizes in those studies were small, with only 1, 19, and 8 long-latency neurons studied by Robertson [23], Brown [24], and Robertson et al. [25], respectively. According to Robertson et al. [25], in both Robertson's [23] and Brown's [24] studies, it is possible that during the process of recording the SGNs, the opening of the cochlea may have selectively affected the OHCs and thus changed the response of the type II neurons. Robertson et al. and Brown were also unable to successfully fill all the neurons with horseradish peroxidase. Consequently, they could not ensure that the neurons were actually type II SGNs [25]. Another line of evidence inconsistent with our observation is that the antidromic response latencies of type II neurons to brainstem shocks are 6-7 ms and are therefore longer than the sound-evoked latencies of MOC efferents, which are 4.5 ms [26]. However, the point of antidromic activation of the central processes can greatly affect latency. For example, the stimulating electrode used by Brown was inserted into the cochlear nucleus, yet the electrode used by Robertson was set on the auditory nerve [25]. As a result, the mean latency for the long-latency neurons recorded by Brown was nearly five times greater than that recorded by Robertson.

Our results support the hypothesis that type II SGNs drive the MOC contralateral suppression reflex. However, the ouabain-exposed mouse model has limitations. Although the morphological evidence showed that there were nearly no type I SGNs in 12 consecutive midmodiolar sections of the ouabain-exposed cochlea, it is possible that the few remaining type I SGNs were able to drive the MOC contralateral suppression reflex. An animal model in which the type II neurons can be manipulated selectively is required for the further exploration of their physiological functions.

## 5. Conclusions

After ouabain exposure, the amplitude of the contralateral suppression was not significantly different from that of the control group. Almost no type I spiral ganglion neurons remained in the ouabain-treated cochleae. Thus, the type II spiral ganglion neurons are almost certainly the afferents responsible for contralateral suppression. Our study helps resolve the controversy about whether type II afferents are driving medial olivocochlear (MOC) efferent fibers [4] or not [6].

## Abbreviations

MOC: Medial olivocochlear
SGNs: Spiral ganglion neurons
OHCs: Outer hair cells
DPOAEs: Distortion product otoacoustic emissions
*Prph*: Peripherin gene
PBS: Phosphate-buffered saline
EDTA: Ethylenediaminetetraacetic acid
OCT: Optimal cutting temperature compound
Tuj1: Tubulin *β*3
Prph: Peripherin protein
ANOVA: Analysis of variance.

## Supplementary Materials

Supplementary Materials Supplemental Table 1: ABR threshold of the sham-operated and ouabain-treated groups (dB SPL). Supplemental Table 2: peak-to-peak amplitude of wave I at 16 kHz in ABR test (*μ*V). Supplemental Table 3: DPOAE threshold of the control group and the surgery group. Supplemental Table 4: amplitude of 2f_1_−f_2_ distortion products at 16 kHz (dB SPL). Supplemental Table 5: density of type I and type II spiral ganglion neurons. Figure S1: contra-noise suppression of DPOAEs disappears after contralateral cochlear destruction (CCD). The right ears were destroyed by opening the cochlear basal turn and then exposed to 76 dB SPL, 13–20 kHz broadband noise (continuous for 15 s, closed field), whereas the DPOAEs were elicited in the left ears with 60 dB SPL, 16 kHz primary tones. All the experimental parameters are the same as we described in the method of the manuscript. *N* = 6, data are presented as the mean ± SEM.
