# Supplementary material for: Contralateral Suppression of DPOAEs in Mice after Ouabain Treatment
Source: Neural Plast. 2018 Apr 10;2018:6890613. doi: 10.1155/2018/6890613 (PMC5914095; doi:10.1155/2018/6890613)
Supplement: Supplementary Materials — Supplemental Table 1: ABR threshold of the sham-operated and ouabain-treated groups (dB SPL). Supplemental Table 2: peak-to-peak amplitude of wave I at 16 kHz in ABR test (μV). Supplemental Table 3: DPOAE threshold of the control group and the surgery group. Supplemental Table 4: amplitude of 2f1−f2 distortion products at 16 kHz (dB SPL). Supplemental Table 5: density of type I and type II spiral ganglion neurons. Figure S1: contra-noise suppression of DPOAEs disappears after contralateral cochlear destruction (CCD). The right ears were destroyed by opening the cochlear basal turn and then exposed to 76 dB SPL, 13–20 kHz broadband noise (continuous for 15 s, closed field), whereas the DPOAEs were elicited in the left ears with 60 dB SPL, 16 kHz primary tones. All the experimental parameters are the same as we described in the method of the manuscript. N = 6, data are presented as the mean ± SEM. [file 6890613.f1.pdf]

The detailed electrophysiological data was as follows. Ouabain gel was applied to the right ears in ouabain treated mice. The right ears of sham operated mice served as the control in our manuscript. The ABR and DPOAE data of the left ears of ouabain treated mice are supplied in the supplemental materials.

Supplemental Table 1. ABR threshold of the sham operated and ouabain treated groups (dB SPL)

Frequency(Hz)	8k	12k	16k	20k	24k
Right ears of Sham operated mice	21±0.3	20±0	22±0.9	21±0.4	25±1.6
Left ears of ouabain treated mice	22±1.0	21±0.4	21±1.1	24±1.3	28±0.3
Ouabain treated ears	52±2.2	54±1.6	59±1.6	64±3	70±3.1

Two weeks after the application of ouabain, the ABR threshold increased significantly compared with that in the sham operated group ($F=529$, $P<0.01$) and left ear ($F=319.5$, $P<0.01$). Two-way ANOVA was used in the analysis. ABR threshold showed no significant difference between the sham operated ears and left ears of ouabain treated ears, $F=3.11$, $P=0.11$.

Supplemental Table 2. Peak to peak Amplitude of wave I at 16 kHz in ABR test (μV)

Level(dB SPL)	40	50	60	70	80
Right ears of Sham operated mice	0.35±0.06	0.57±0.16	0.99±0.32	1.36±0.33	2.22±0.54
Left ears of ouabain treated mice	0.39±0.14	0.65±0.14	0.96±0.35	1.28±0.53	1.94±0.65
Ouabain treated ears	-	-	0.098±0.03	0.19±0.08	0.26±0.08

Two weeks after the application of ouabain, the ABR threshold increased significantly compared with that in the sham operated ear ($F=30.78$, $P<0.01$) and left ear of ouabain treated mice ($F=71.61$, $P<0.01$). Amplitude of wave I showed no significant difference between the sham operated ears and left ears of ouabain-treated mice ($F=0.29$，$P=0.59$). Two-way ANOVA was used in the analysis.

Supplemental Table3. DPOAE threshold of the control group and the surgery group

Frequency	8k	12k	16k	20k	24k
Right ears of Sham operated mice	41±1.1	38±1.4	29±0.6	45±1	57±1.2
Left ears of ouabain treated mice	50±1.4	42±0.8	29±0.6	46±1.3	53±1.2
Ouabain treated ears	49±2	49±1.9	31±0.9	43±1.8	46±1.8

Ouabain treatment had no significant effect on the threshold of DPOAEs compared with the sham operated ears and the left ears of ouabain treated mice, $F=0.37$, $P=0.69$. (Two-way ANOVA)

Supplemental Table 4. Amplitude of $2f_1 - f_2$ distortion products at 16 kHz (dB SPL)

Level(dB SPL)	40	50	60	70	80
Right ears of Sham operated mice	23±0.9	30±1.6	38±0.8	42±0.9	49±0.8
Left ears of ouabain treated	20±1.1	29±1.5	38±1.4	44±1.4	48±0.9

mice					
Ouabain treated ears	21±1.6	29±1.6	36±1.4	41±1.3	48±1.1

Ouabain treatment had no significant effect on the amplitude of DPOAEs at 16 kHz compared with the sham operated ears and the left ears of ouabain treated mice, F=0.53, P=0.59. (Two-way ANOVA)

Supplemental Table 5. Density of type I and type II spiral ganglion neurons

	Type I SGNs(/mm^2)	Type II SGNs(/mm^2)
Right ears of Sham operated mice	1898±103	202±58
Left ears of ouabain treated mice	2048±122	213±67
Ouabain treated ears	10±6	190±62

After the ouabain treatment, the density of type I spiral ganglion decreased significantly compared with sham operated ears (P<0.01, one-way ANOVA) and left ears of ouabain treated ears (P<0.01, one-way ANOVA) . However, ouabain treatment had no significant effect on the density of type II spiral ganglion, P=0.95(one- way ANOVA).

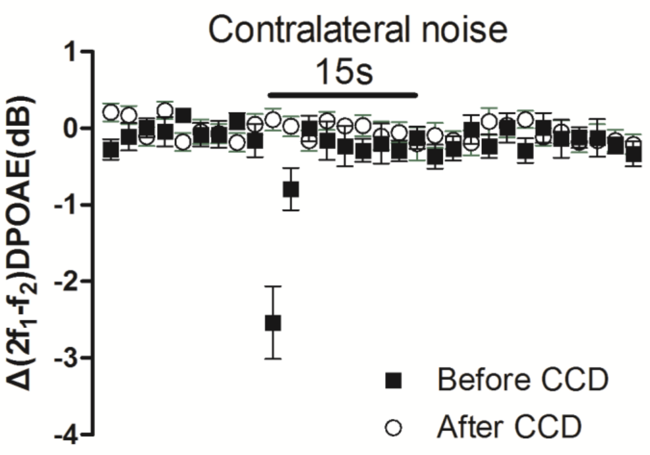

Figure S1 Contra-noise suppression of DPOAEs disappears after contralateral cochlear destruction (CCD). The right ears were destroyed by opening the cochlear basal turn and then exposed to 76dB SPL, 13–20 kHz broadband noise (continuous for 15 s, closed field), whereas the DPOAEs were elicited in the left ears with 60dB SPL, 16kHz primary tones. All the experimental parameters are the same as we described in the method of the manuscript. N=6, data are presented as mean±SEM.
